# Evaluation of backscatter dose from internal lead shielding in clinical electron beams using EGSnrc Monte Carlo simulations

**DOI:** 10.1120/jacmp.v16i6.5563

**Published:** 2015-11-08

**Authors:** Rowen J. de Vries, Steven Marsh

**Affiliations:** ^1^ Department of Radiation Oncology Palmerston North Hospital Palmerston North New Zealand; ^2^ Department of Physics University of Canterbury Christchurch New Zealand

**Keywords:** backscattered electrons, Monte Carlo simulation, electron radiotherapy, dosimetry, scattered radiation, internal lead shielding

## Abstract

Internal lead shielding is utilized during superficial electron beam treatments of the head and neck, such as lip carcinoma. Methods for predicting backscattered dose include the use of empirical equations or performing physical measurements. The accuracy of these empirical equations required verification for the local electron beams. In this study, a Monte Carlo model of a Siemens Artiste linac was developed for 6, 9, 12, and 15 MeV electron beams using the EGSnrc MC package. The model was verified against physical measurements to an accuracy of better than 2% and 2 mm. Multiple MC simulations of lead interfaces at different depths, corresponding to mean electron energies in the range of 0.2–14 MeV at the interfaces, were performed to calculate electron backscatter values. The simulated electron backscatter was compared with current empirical equations to ascertain their accuracy. The major finding was that the current set of backscatter equations does not accurately predict electron backscatter, particularly in the lower energies region. A new equation was derived which enables estimation of electron backscatter factor at any depth upstream from the interface for the local treatment machines. The derived equation agreed to within 1.5% of the MC simulated electron backscatter at the lead interface and upstream positions. Verification of the equation was performed by comparing to measurements of the electron backscatter factor using Gafchromic EBT2 film. These results show a mean value of 0.997±0.022 to 1σ of the predicted values of electron backscatter. The new empirical equation presented can accurately estimate electron backscatter factor from lead shielding in the range of 0.2 to 14 MeV for the local linacs.

PACS numbers: 87.53.Bn, 87.55.K‐, 87.56.bd

## I. INTRODUCTION

Electron beam therapy (EBT) can be the preferred method of treatment for several superficial treatment sites of the head and neck. One of the benefits of EBT is the sharp dose gradient beyond the therapeutic depth sparing the deeper healthy tissue. Dose to deep tissues can be further reduced in certain situations by introducing shielding. Pb shielding can be placed internally in treatments of the lip, eyelids, buccal mucosa, and the nose. Space permitting, the thickness of this Pb layer is optimized to the mean electron energy at the Pb/tissue interface to achieve the required dose reduction.^(1)^

It is well known that Pb will cause some electrons to undergo multiple elastic Coulomb scattering processes resulting in some electrons tracking back towards the source.^(2)^ These electrons are known as backscattered electrons. The backscattered electrons are absorbed into the upstream tissue and this impact on the dose distribution must be considered. Inaccuracies in this upstream dose enhancement can cause unaccounted intermediate or late complications for the patient.^(3)^

### A. Electron backscatter factor

The dose enhancement due to backscattered electrons from the Pb interface can be quantified by the electron backscatter factor (EBF). The EBF is defined as the ratio of dose at the Pb interface to the dose at the same point in the absence of Pb.^(4)^ It is usually quantified on the central axis of the electron beam. This definition makes the EBF value a point quantity which is physically difficult to measure using volumetric devices such as ion chambers.^(5)^

**Figure 1:**
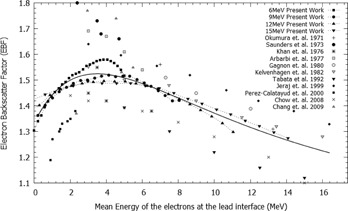
Various relative electron backscatter factors from a Pb interface for several different energies calculated from the MC simulation and compared to various electron backscatter factors from other works^4^, ^5^, ^8^, ^9^, ^10^, ^11^, ^12^, ^13^, ^14^, ^15^ and Eq. (7) (line).

Several previous studies have measured and quantified the electron backscatter factor for various treatment machines using different radiation detectors or Monte Carlo (MC) methods. A list of the most notable experimental results published can be found in Pérez‐Calatayud et al.^(5)^ Results for several of these studies are plotted in Fig. 1. From this figure, a clear relationship of decreasing EBF with increasing mean energy at the lead interface is observable above 4 MeV; however, the relationship is not as clear for energies below a mean energy of 4 MeV at the lead interface.

Of particular significance is the work of Klevenhagen et al.^(4)^ who comprehensively investigated EBF values for several materials and depths using a purpose built ionization chamber. Measurements were made using a range of energies from 3 to 35 MeV produced by a 20 MeV linac (SL 75/20, Philips, UK) and a 35 MeV betatron (BBC betatron Asklepitron 35; Brown Boveri Corp., Zurich, Switzerland). From their work, the following relationship between the EBF and the mean energy at the interface was determined:

(1) $$EBF=1+0.735\times {\text{exp}}^{-0.052E_{m}(z)}$$

where $E_{m}$ is the mean energy in MeV at the Pb interface at a depth of *z* mm and can be approximated using a variant of Equation 2.25 from ICRU Report 35:^(6)^

(2) $$E_{m}\approx E_{0}(1-z/R_{p})$$

where $R_{p}$ is the practical range of the electron beam, and $E_{0}$ is the mean energy at the surface. Equation (1) agreed to their experimental data to within 2.9% and is currently assumed to be the best model for estimating EBF values for clinical purposes. However, Eq. (1) is, on occasions, used outside the range over which it is valid (3 to 35 MeV).

### B. Electron backscatter intensity

The dose enhancement upstream from the Pb shielding interface is also of clinical importance. Electron backscatter intensity (EBI) is defined as the relative electron backscatter intensity upstream from the Pb interface in relation to the amount of EBF at the Pb interface, and is a metric for quantifying the upstream dose enhancement.^(7)^ Since backscatter electrons are predominantly produced through multiple elastic scattering events, they can penetrate substantially in the upstream direction (e.g., 10–20 g/mm in polyester).^(7)^

The upstream EBI attributed to by the backscattered electrons has been quantified by Lamber and Klevenhagen^(7)^ who measured EBI values over a range of electron energies from 1 to 25 MeV. From their work, the following relationship for the EBI was determined:

(3) $$EBI(t)=A\times {\text{exp}}^{-kt}$$

where *t* is the distance upstream from the interface in mm, the constants *A* and *k* are dependent on the primary electron beam energy and can be found in Lambert and Klevenhagen.^(7)^

However, evidence in the literature^4^, ^5^, ^8^, ^9^, ^10^, ^11^, ^12^, ^13^, ^14^, ^15^ suggests that there may be a different relationship for EBF and EBI in the energy region below 4 MeV. This corresponds to the energy of electrons often present at the Pb interface during treatments utilizing 6 and 9 MeV electron beams. More recently, physical measurements and MC studies have shown a trend of decreasing EBF with decreasing energy in the lower energy region. Pérez‐Calatayud et al.^(5)^ proposed that the EBF increases with energy in the region of 0.5 to 1.5 MeV and then remains nearly constant up to 3 MeV (the upper energy limit of their study). Their results disagree with Eq. (1) when extrapolated to energies below 3 MeV.

Furthermore, previous literature^4^, ^5^, ^8^, ^9^, ^10^, ^11^, ^12^, ^13^, ^14^, ^15^ shows a large spread of EBF values which were obtained from a range of conventional dosimeters such as film, ion chambers, MOSFETs, TLDs, and MC simulations. Several of the authors noted difficulty in measuring the EBF value because of its point quantity definition. Many of the studies indicated errors of between 5% and 20%. This uncertainty in the data could explain the spread of measured values; however, several authors also attribute this spread to the different electron spectra produced by different electron beam generating machines.

The aim of this work is to use MC method to evaluate the accuracy of the empirical EBF and EBI equation for the local electron beams. The local treatment machines will be simulated over the entire range of electron energies clinically available. It is anticipated that a single equation will be derived that will be able to estimate both the EBF and EBI value to a clinically acceptable accuracy for these electron beam.

## II. MATERIALS AND METHODS

The latest version (4‐r 2.3.1) of EGSnrc^(16)^ family of MC code from National Research Council of Canada at time of publication was used throughout this work. The linac head model and phase space files were simulated using BEAMnrc (v2.0).^(17)^ The dose distributions were calculated using DOSXYZnrc (v1.1)^(18)^ along with the linac head phase space files.

### A. Monte Carlo linac simulation

The graphical user interface version of BEAMnrc code was used to simulate the treatment head of a Siemens Artiste linac (Siemens Oncology Care, Erlangen, Germany). Electron beams with nominal energy of 6, 9, 12, and 15 MeV were simulated. A selection of electron applicator sizes were chosen for verification (100, 150, 200 mm squares, 50 mm diameter circle and without an applicator). Simulating a large range of cone sizes will give confidence in the electron fluence of the model. The specifications of the physical dimensions and material composition of the treatment head were obtained from the manufacture under a nondisclosure agreement and will not be reproduced here.

Simulation parameters in BEAMnrc were largely unchanged from the default settings. The Parameter Reduced Electron‐Step Transport Algorithm II (PRESTA‐II) was used with the parameters set to default. The transport parameters were set to ECUT = 700 keV, PCUT = 10keV, and ESTEP = 0.25, remembering that the value of ECUT includes the rest mass of an electron (511 keV). The initial mono‐energetic energy of the primary electrons was first estimated using $E_{0}=C\times R_{50}$, where $C=2.33\;{\text{MeV}}^{-1}$ and $R_{50}$ is in cm.^(6)^ This was iteratively adjusted until the resulting percentage depth dose (PDD) curves and the measured data agreed within 2%. 500 million histories were simulated in the final phase space file which was scored at an isocentric plane for each electron beam. On average, each phase space file contained 90 million particles with statistical uncertainty below 0.2% over the scoring plane. This, along with other information regarding the phase space files can be found in Table 1.

**Table 1:** Initial beam parameters and simulation results for a BEAMnrc MC simulation of a Siemens Artiste for 6, 9, 12, and 15 MeV electron beam for a 10 cm square electron applicator

*Nominal Beam Energy (MeV)*	*6*	*9*	*12*	*15*
Peak energy of energy spectrum (MeV)	6.40	9.40	12.49	16.15
FWHM of energy spectrum (mm)	0.21	0.30	0.30	0.25
Number of participles in phase space file (×106)	93.0	99.0	102.0	110.0

### B. Monte Carlo verification

The DOSXYZnrc code was used to calculate dose planes using the phase space files on a 40 cm cube water phantom. The calculation grid was set to 1 mm in size. The number of particles simulations was set to 200 million histories. The recycling rate was set according to the number of particles in the phase space file to maintain counting statistics. Depth‐dose curves and beam profiles were extracted and normalized to the maximum dose value ($D_{\text{max}}$) on the central axis.

Verification of the model was performed via comparison of central axis PDD curves and beam profiles with physical measurement. Physical measurements were acquired using a waterproof 3G‐pSi Scanditronix‐Wellhofer electron diode radiation field detector (Scanditronix‐Wellhofer Dosimetry, IBA, Schwarzenbruck Germany) in a scanning water phantom and OmniPro 7.3 acquisition software (Blue Phantom 2, IBA). Central axis PDD curve and beam profile measurements for electron beams with energies of 6, 9, 12, and 15 MeV were acquired using a range of electron applicators identical to those modeled.

### C. Backscatter simulations

The verified BEAMnrc simulation phase space files were used to perform electron backscatter simulations using DOSXYZnrc. The calculation grid was set to 1 mm in size and the number of particles simulations was set to 200 million histories. Simulations of a number of Pb interface depths were performed with the interface positioned at depths according to Table 2. The mean electron energy at the Pb surface was estimated using Eq. (2). This range of Pb depths resulted in a range of mean electron energies at the Pb surface from 0.2 to 14.0 MeV. The Pb thickness was specified sufficiently thick to provide saturation levels of backscatter for each depth position and energy.

Central axis PDD curve were extracted for each simulation and were used to calculate the EBF and EBI values. These values were taken as the ratio of dose between the simulation with and without a Pb interface. The calculated EBI values were attributed to the center of each voxel, and EBF values were attributed to the center of the first upstream voxel from the Pb interface.

**Table 2:** Depths at which a Pb slab was positioned in MC simulations of backscatter using DOSXYZnrc

*Energy*	*Depth Range (mm)*	*Interval (mm)*
6 MeV	3–29	1
9 MeV	3–45	2
12 MeV	3–60	3
15 MeV	4–76	4

### D. Experimental measurements

In order to validate the empirical equation derived from the EBF simulations, measurements were made using Gafchromic EBT2 film (Lot number A10061001B; International Specialty Products, Wayne, NJ) inside a slab phantom of virtual water. Utilizing virtual water decreases the positioning uncertainty when compared to performing measurements in water. The energy dependence of virtual water has been shown to be in the range of 0.1% to 0.4% over an energy range of 4 to 22 MeV at and around the depth of dose maximum ($d_{\text{max}}$).^(19)^ This is an acceptable uncertainty.

Calibration of the Gafchromic film was performed in virtual water for each beam energy at its respective reference depth. The film was scanned using an Epson V700 scanner (US Epson, Long Beach, CA). An average calibration curve for the red channel was acquired. The difference in pixel value between individual calibration points and the calibration curves were not larger than ± 0.8% which translates into a ± 2.4% uncertainty in dose.

EBF film measurements were made in succession at the same depth in virtual water, with and without the Pb present. A Pb sheet 5 mm in thickness was used to provide the backscattered electrons, and was positioned at depths of 10, 20, 30, and 40 mm for all energy electron beams except 6 MeV (10 and 20 mm only). This resulted in a range of 14 different mean electron energies at the Pb interface ranging from 0.8 to 13.2 MeV, calculated using Eq. (2). EBT2 film was positioned at 0, 1, 2, 5, and 7 mm upstream from the position of the Pb interface.

The backscatter value was taken as the ratio of these measurements and a range scaling correction factor was applied to the results. The range scaling correction factor converts a depth in virtual water to the equivalent depth in water. This was measured for each energy electron beam by direct comparison of partial depth ionization curves obtained in both virtual water and water. Measurements were acquired using a Roos chamber (PTW Roos chamber type 34001; Freiburg, Germany) for both the virtual water phantom and small water tank. The resulting mean range scaling correction factor was 1.02. This compares well with the range scaling factor measured by McEwen and Niven^(19)^ of 1.01.

## III. RESULTS

### A. MC linac simulation

**Figure 2:**
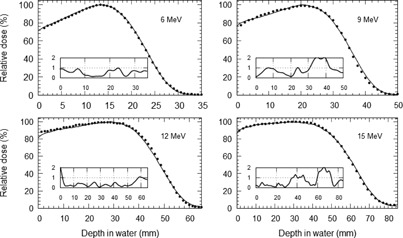
Depth‐dose curve comparison between the BEAMnrc MC simulations (points) and water phantom measurements (lines) for 6, 9, 12, and 15 MeV electron beams with absolute percentage difference between the model and measurement shown in the inset.

Figure 2 shows the results of the MC simulation compared with PDD measurements for a 10 cm square electron field. The MC PDD curves agreed with the measured PPD curves to within 2% from the surface to depths of $R_{p}$. Other field sizes showed comparable results and are not reproduced here.

**Figure 3:**
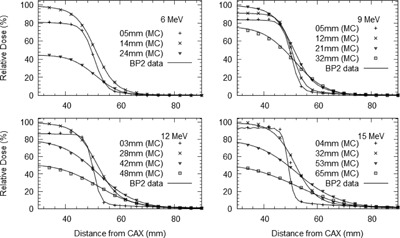
Profile comparison between the BEAMnrc MC simulations and water phantom measurements (lines) for 6, 9, 12, and 15 MeV electron beams for a 10 cm square electron applicator at different depths in water.

Figure 3 shows the results of the MC simulation compared with profile measurements for a 10 cm square electron field for all energies. The agreement of the profiles was good, including the penumbral regions of the fields. Other field sizes showed comparable results and are not reproduced here. A comparison of measured and simulated results for an electron beam without an electron applicator was also made for 6 MeV with good results. Comparing simulated and measured data for several different electron applicator field sizes, and without electron applicator altogether, gives confidence in the electron spectrum produced by the model.

### B. MC electron backscatter simulations

**Figure 4:**
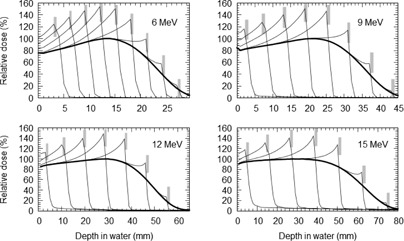
Various percentage depth‐dose curves for different energy electron beams with Pb positioned at different depths compared to the standard PDD curves (bold curve), clearly showing the electron backscatter effect. The Pb positions are represented by the gray bars.

**Figure 5:**
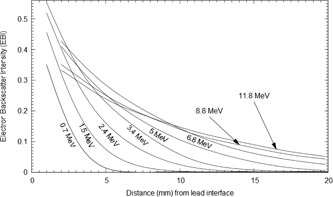
Various relative electron backscatter intensities in the upstream direction from a Pb interface averaged over several different energy regions calculated from MC backscatter simulations.

The resulting PDD curves from the MC simulations for several of the Pb positions normalized against the standard PDD curve are shown in Fig. 4. From these curves, the EBF and EBI values were calculated for each Pb depth position. The EBF values were determined using the dose to the first voxel upstream from the Pb interface and attributed to its centre. The EBF values obtained are plotted in Fig. 1 as a function of the mean electron energy at the interface. The results are also compared to various EBF values from other works in the literature. EBI curves were averaged for a number of different mean energy ranges. The upstream EBI curves for different mean energy ranges are shown in Fig. 5.

The EBF results in Fig. 1 show that the relationship between the EBF and mean energy at the Pb interface cannot be empirically estimated by an equation of the form in Eq. (1). On average, Eq. (1) overestimates the EBF value by 11.6% in the energy range of 1 to 5 MeV, with a maximum overestimation of 31% when compared to the MC results. The agreement in the energy range above 5 MeV is much better and, on average, overestimates the EBF value by 3.8%. The EBI results in Fig. 5 were also compared to empirical curves calculated from Eq. (3). The disparity between Eq. (3) and the MC results is most evident in the lower energy range.

A new empirical equation was derived from the data to allow estimation of both the EBF and EBI for the local electron beams. This equation reflects the decreasing EBF and EBI seen at low energies, as shown by the results. The electron backscatter factor as a function of distance from the interface t (EBF(t)) was defined as the ratio of dose at a distance, t, in mm which is at, or upstream from, a Pb interface, to the dose at the same depth without the Pb interface present under saturation electron backscatter conditions. This combines both EBF and EBI into one term. An equation was first derived to estimate EBF as a function of the mean energy at the Pb interface and was found to be describable by the following relationship:

(4) $$EBF(E_{m})=1+C_{1}{\text{exp}}^{-C_{2}E_{m}}-C_{3}{\text{exp}}^{-C_{4}E_{m}}$$

where $E_{m}$ is the mean energy in MeV at the Pb interface, $C_{1-4}$ are experimentally determined constants with values of 0.936±0.132, $0.089\pm 0.012\;{\text{MeV}}^{-1}$, 0.602±0.126, and $0.375\pm 0.065\;{\text{MeV}}^{-1}$ to within a 95% confidence level respectively. The root mean square error between Eq. (4) and the measured values was 0.024. These coefficients are specific to the Siemens Artiste electron beams and should be verified for other machines; however, Eq. (4) is representative of the trend shown in the previously measured EBF values from other machines' electron beams (Fig. 1).

**Figure 6:**
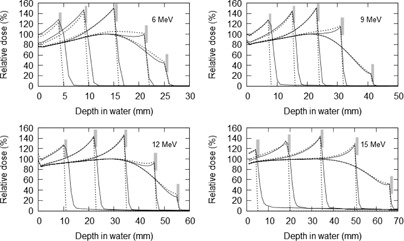
Comparison of percentage depth‐dose curves involving Pb calculated using Eq. (7) (dashed line) and simulated using Monte Carlo (solid line) for a selection of different Pb depths. The Pb positions are represented by the gray bars.

The overall equation for EBF(t) combines Eq. (4) and an exponential decay factor:

(5) $$EBF(E_{m},t)=EBF(E_{m}){\text{exp}}^{-k(E_{m})t}$$

where *t* is the distance upstream from the interface in mm and *k* is described by the logarithmic expression

(6) $$k(E_{m})=-C_{5}\text{ln}(E_{m})+C_{6}$$

where $C_{5}$ has a value of $0.130\pm 0.004\;t^{-1}$ and $C_{6}$ has a value of $0.413\pm 0.006\;t^{-1}$ to within a 95% confidence level. Combining Eqs. (4), (5), and (6) results in the final expression for EBF(t), shown in Eq. (7). The resulting equation is one that can be used to estimate the dose enhancement cause by backscattered electron from Pb at the depth of the interface, or upstream for any mean electron energy at the interface in the range of 0.2 to 14.0 MeV

(7) $$EBF(E_{m},t)=1+(C_{1}{\text{exp}}^{-C_{2}E_{m}-C_{6}t}-C_{3}{\text{exp}}^{-C_{4}E_{m}-C_{6}t})\times E_{m}{\;}^{-C_{5}t}$$

where $E_{m}$ is in MeV and *t* is in mm. Equation (7) agrees with the MC simulated EBF values at the Pb interface with an average percentage difference of 1.3% and a maximum percentage difference of 4.9%. Comparisons of the MC and equation predicted EBF(t) were made for energies identical to those in Fig. 5 and the average percentage difference was 1.5% for EBF(t) values greater than 1.05. Figure 6 shows the comparisons of PDD curves estimated using Eq. (7) and the MC results for a selection of different Pb interface depths and initial energy electron beams.

### C. Gafchromic film results

**Figure 7:**
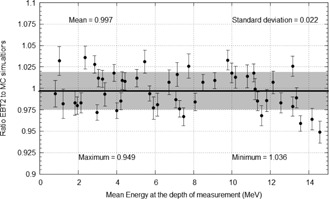
Graph of ratios between electron backscatter values measured using EBT2 film and estimated using Eq. (7). The gray band representing 1 SD from the mean of 0.997.

Gafchromic film measurements were used to verify the accuracy of Eq. (7). The ratio of EBF(t) values measured using Gafchromic film and estimates from Eq. (7) are shown in Fig. 7. The results show a mean ratio of 0.997±0.022 to 1σ.

The largest deviation between the film result and Eq. (7) was 5.12%. This deviation was seen in the measurement of backscatter from a 15 MeV electron beam with Pb positioned at a depth of 10 mm and film position at 7 mm upstream from the Pb interface. This large difference is most likely due to the insufficient thickness of Pb to provide saturation electron backscatter for this particular mean energy at the Pb interface compared to the MC simulations. The other upstream measurement points for this particular set up also showed differences in the order of 3%–4 %. When this measurement set is excluded, the ratio between film measurements and Eq. (7) improves to 1.000±0.019 to 1σ. Furthermore, the largest deviation decreases to a value of 3.6%.

**Figure 8:**
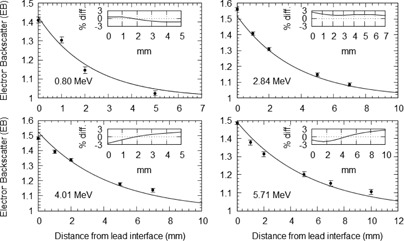
Graph comparing measured (points) and calculated (lines) relative upstream electron backscatter from a Pb interface for several electron beams with mean energies of 0.8o, 2.84, 4.01, and 5.71 MeV. Measurements were made using Gafchromic film in virtual water; calculations were performed using Eq. (7).

Figure 8 plots results for a series of measured EBT(t) values and compares them to Eq. (7). The results of which were good with a maximum difference of 2.9%.

## IV. DISCUSSION

The simulation and measurement results of the EBF confirm the relationship between EBF and the mean energy at the Pb interface. EBF first increases with increasing energy up until a maximum is reached. Beyond this, the EBF decreases more slowly with further increases of energy. The results established that EBF is independent of beam energy for 9, 12, and 15 MeV electron beams for the Siemens Artiste linac. The EBF values obtained for these energies are largely within 1% of each other for the same energy at the interface. However, the EBF values from the 6 MeV simulations deviated by up to 5% above 4 MeV. The reasons behind this is not fully understood, and may be due to a difference in electron angular spread and energy spectra caused by the absence of a primary scattering foil for this energy in this particular linac model, whereas all other energies use both a primary and a secondary scattering foil. This may produce an insufficient spread in the energy fluence of electrons to allow the mean electron energy parameter to be a good descriptor of EBF for the 6 MeV beam.

The difference may also stem from the use of Eq. (2) to calculate the mean energy. This equation was derived from data using an almost mono‐energetic 20 MeV electron beam incident on a carbon absorber. Therefore, Eq. (2) may not be applicable to the local electron beams in water and more work is required in verifying this.

Conversely, the larger EBF values above a mean energy of 4 MeV could be related to the increase of the photoelectric effect in Pb for the low‐energy photon contamination component of the beam. This effect would decrease with decreasing mean energy at the interface, as the low‐energy photons are absorbed as they pass through the water before reaching the Pb interface. The values obtained from the 6 MeV simulations show this behavior. Below a mean energy of 4 MeV at the interface, the results agree to within 1% of the other energy beams.

The EBF values calculated in this work through MC simulations of a Siemens Artiest are specific to a Siemens Artiests, however they display a similar trend and are centered within previously acquired data from various linacs. This highlights the issue of using Eq. (1) below 4 MeV which is likely present, and should be considered, with all linacs

Comparing Eq. (7) to previous data showed an average difference of 0.4%, but with a large standard deviation of 9.4%. The centrality of the data can be seen when comparing the difference to datasets calculated by Jaraj et al.,^(14)^ which were higher (7.9%), and by Tabata and Ito,^(11)^ which were lower (−8.8%). Similarly, the data from Chow and Grigorov^(8)^ showed a difference of 7.2%. Equation (7) does overestimate the EBF in the low treatment energy region below 3 MeV consistently by 10% when compared to data from Pérez‐Calatayud et al.^(5)^

**Figure 9:**
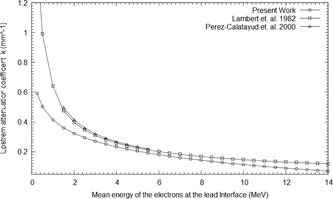
Comparison of upstream electron backscatter decay coefficients for different mean energies of electrons at the Pb interface between the present work and existing equations proposed by Lambert and Klevenhagen^(7)^ and Pérez‐Calatayud et al.^(5)^

The decay of EBT calculated by Eq. (5) can be compared to previous empirical expressions by Lambert and Klevenhagen^(7)^ and Pérez‐Calatayud et al.^(5)^ through the decay coefficient k. Figure 9 compares the decay coefficient calculated by Eq. (6) to these expressions over their valid energy ranges. This figure shows that the decay of the upstream dose enhancement presented here is slower than these previous expressions, which indicates a longer range of the backscattered electron for the local electron beams.

Equation (7) may prove useful for clinical dosimetric planning involving Pb internal shielding in electron beam therapy for a wide range of treatment machines. The match of Eq. (7) to previous data obtained from several different electron beams affirms this. However, the equation and coefficients calculated here are specific to Siemens linac electron beams and should be verified for other electron beams.

## V. CONCLUSIONS

A new empirical equation was derived to estimate the electron backscatter factor at a distance t from Pb shielding (EBF(t)) during electron beam treatments with a Siemens Artiste linac. This was achieved from MC simulations of Pb interfaces in water at different depths and extracting EBF(t) values. Pb interfaces were positioned at several depths in water to vary the mean energy at the Pb interface; this resulted in a range of mean energies from 0.2 to 14 MeV. This energy range adequately covers the range of typical energies expected at the interface in clinical situations.

The proposed equation agreed to MC data to an average percentage difference of 1.5% both at the interface and at upstream positions. This equation also follows a similar relationship to previously measured EBF(t) values in the literature. That is, the electron backscatter first increases with increasing energy until 4 MeV, then, with further increasing energy, the electron backscatter decreases exponentially.

Verification of the derived equation was performed though a comparison measurement with Gafchromic film in a Solid Water phantom. The results showed agreement of ± 2.2% to 1σ, which is acceptable for clinical estimation of the intensity of electron backscatter and represents a better fit than the previously used equation for the local treatment machines.
